# An Occupational Heat Stress and Hydration Assessment of Agricultural Workers in North Mexico

**DOI:** 10.3390/ijerph17062102

**Published:** 2020-03-22

**Authors:** Rietta S. Wagoner, Nicolas I. López-Gálvez, Jill G. de Zapien, Stephanie C. Griffin, Robert A. Canales, Paloma I. Beamer

**Affiliations:** 1 Department of Community, Environment, and Policy, Mel and Enid Zuckerman College of Public Health, University of Arizona, Tucson, AZ 85721, USA; lopezgalvez@email.arizona.edu (N.I.L.-G.); scgriffin@email.arizona.edu (S.C.G.); pbeamer@email.arizona.edu (P.I.B.); 2 Department of Health Promotion Sciences, Mel and Enid Zuckerman College of Public Health, University of Arizona, Tucson, AZ 85721, USA; dezapien@email.arizona.edu; 3 Interdisciplinary Program in Applied Mathematics, University of Arizona, Tucson, AZ 85721, USA; rcanales@email.arizona.edu

**Keywords:** climate change, dehydration, farm worker, heat strain, thermal stress

## Abstract

Expanding agribusiness in Northern Mexico has increased demand for workers from Southern Mexico, with hundreds of thousands migrating for work annually. Extreme temperatures, physical labor, and low fluid consumption place workers at risk for heat strain and dehydration, commonly underreported hazards in the agricultural industry. The objectives of this pilot study were to assess heat exposure and hydration status of a population of migratory agricultural workers in Northern Mexico throughout the grape harvest season. In addition to demographic information, environmental conditions, hydration status, and core body temperatures were collected. The majority listed Chiapas as their home state, nearly half spoke an Indigenous language, and none had completed high school. The wet-bulb globe temperature was significantly higher during the harvest and post-harvest seasons compared to the pre-harvest season. Across the different seasons, the majority were dehydrated post-shift, and mean core body temperature of workers was not significantly different. This project highlights the need for targeted interventions to improve hydration and prevent heat stress in this region. As the number of warm days is expected to rise each year worldwide, it will be increasingly important to engage in practices to protect vulnerable populations, such as migratory agriculture workers.

## 1. Introduction

Agricultural workers participate in strenuous tasks and experience an array of occupational risks and hazards. High humidity and extreme ambient temperatures coupled with heavy physical labor and low fluid consumption place individuals at risk for heat strain and dehydration, serious and commonly underreported hazards in the agricultural industry [1,2,3,4,5]. As the number of hot days and heatwaves is expected to increase globally, it is expected that vulnerable populations, such as agricultural workers, will experience increased heat-related morbidity and mortality [1,6,7,8,9,10,11,12].

The Northwestern state of Sonora, Mexico comprises approximately 95% arid or semi-arid land characterized by lack of precipitation and high temperatures [13,14]. Expanding agribusiness in Sonora has increased the demand for temporary migrant agricultural workers from Southern Mexico, with hundreds of thousands of individuals migrating to the region each year for agricultural work [15,16]. Migrant families are recruited and contracted in their hometowns and travel by bus from some of the poorest regions in Chiapas, Oaxaca, Puebla, Guerrero, and Veracruz [17]. Due to their adverse socioeconomic status, migratory and seasonal agricultural workers are more likely to accept and remain in high-risk jobs, making them a vulnerable group [15,18]. Between 2002 and 2010, Mexico recorded 393 heat-related deaths, mainly in the northwestern region of the country that includes the state of Sonora [19]. Over a third of these deaths were agricultural workers who had migrated from Southeastern Mexico [20].

Exposure to heat can cause a range of adverse health effects including damage to major organs and even death if the core temperature of the body exceeds 42 °C [11,21,22,23,24]. Recent work has emphasized the deleterious effects heat exposure can have on workers from a productivity standpoint, with reduced productivity as heat exposure increases [3,11,24,25,26,27]. Furthermore, agricultural workers frequently make self-care decisions based on the perception of productivity losses or gains, resulting in higher rates of heat-related injuries when paid by the pound, piece, or line of work completed in the field [28,29,30]. Heat strain can be prevented with adequate fluid intake, sufficient resting periods in shaded areas, adequate time for recovery in cooler areas, the introduction of increased convective and evaporative cooling, and appropriate clothing such as nanoporous metalized polyethylene textiles or cooling vests [31,32,33,34]. Hydration status plays an important role in heat strain; as dehydration progresses, there is a possibility for impairment of the body’s thermoregulatory process, potentially allowing for dangerous increases in core body temperature [31]. Furthermore, elevated dehydration and core body temperatures are associated with decreased physical and cognitive functioning, both of which may contribute to increased risk for injury [35]. The International Organization for Standardization (ISO) has developed screening methods and guidelines to prevent core body temperature from passing a 38 °C threshold [36]. Similarly, the American Conference of Governmental Industrial Hygienists (ACGIH) has set a Threshold Limit Value (TLV) for core body temperature at 38 °C to prevent heat stress in working populations, with the goal of maintaining a body core temperature within +1 °C of 37 °C [37]. 

To the best of our knowledge, no investigations to date have examined both exposure to heat stress and hydration status in migratory farmworker populations in Sonora, Mexico. Therefore, the objective of this pilot study was to assess the heat and hydration status of a vulnerable occupational group at different points during the growing season in Northern Mexico. Furthermore, this project aimed to describe the demographics and occupational history of a largely invisible population in order to provide effective recommendations and inform future study and intervention design.

## 2. Materials and Methods

### 2.1. Study Population

We conducted a cross-sectional study to evaluate heat exposure and hydration of farmworkers. Migratory farmworkers were recruited from a commercial grape farm near Hermosillo, Mexico in March, June, and August 2016 to capture the pre-harvest, harvest, and post-harvest seasons, respectively. During the pre-harvest season in March, workers trim and shape young grape plants to prepare the plants for optimal production. In the June harvest season, workers cut grape bunches from the plant and then weigh and package the fruit for sale. Post-harvest, in the month of August, workers complete a myriad of tasks, from clearing fields to driving tractors. The participants were recruited at the end of each workday with the assistance of the farm physician and engineering teams. A bilingual investigator screened potential participants in Spanish and obtained written consent for participation in the project. The sampling procedure was explained to participants in Spanish and all sampling equipment was shown to participants to ensure they were comfortable with the sampling methods. At the conclusion of the field study, each participant received a small monetary compensation. The individuals that participated in March were included in the June assessment. By August, the majority of workers from the original March cohort had returned to their home state or had transferred to a different farm. As such, a new group of participants was recruited in August. Before the initiation of the study, all participant and data-related methodology was approved by the University of Arizona Human Subjects Protection Program.

### 2.2. Questionnaire

In the months of June (harvest season) and August (post-harvest season), participants completed a questionnaire administered orally in Spanish at the conclusions of the workday. The questionnaire included questions regarding demographic information, work history, and worker-reported heat and hydration experiences including experiences from the day of sampling.

### 2.3. Heat Assessment

Workers were given an ingestible core body sensor to swallow (CorTemp Ingestible Core Body Temperature Sensor, HQ Inc., Palmetto, FL, USA) and assigned a core body temperature monitor to wear around the waist to receive the sensor signals throughout the workday (Wireless Core Temperature Monitoring Data Recorder, HQ Inc., Palmetto, FL, USA). The monitors were set to record temperatures every 10 s and were checked periodically throughout the workday by the investigators.

Following ACGIH guidelines, clothing, metabolic rate, and the natural wet-bulb temperature, globe temperature, and dry-bulb temperature of the environment were measured and incorporated in the Wet-Bulb Globe Temperature (WBGT) calculation to assess workers’ heat exposure. WBGT measurements were taken in the sun and in the shade using a handheld electronic heat stress monitoring device periodically throughout the day (HT30 Heat Stress WBGT Meter, Extech, Nashua, NH), recorded in a field notebook, and later transcribed into an electronic spreadsheet. The handheld WBGT device was carried by the investigators through the fields to ensure that measurements were taken in the immediate work areas during the workday. Observations about metabolic rate and clothing were recorded. To estimate the metabolic rate, observations of individual worker arm movement, leg movement, working speed, trunk movement, pushing and lifting, tool usage, and lateral and vertical movement were recorded. The metabolic rate, measured in watts (W), was then estimated using the ACGIH Table for Metabolic Rate Categories and the Representative Metabolic Rate with Example Activities [37]. Clothing type was categorized according to the ACGIH Clothing Adjustment Factors for Some Clothing Ensembles and added to the effective WBGT (WBGT_eff_). 

### 2.4. Hydration Assessment

Post-shift and morning void urine samples were collected, refrigerated on-site, and transported in ice chests to the University of Arizona within 24 h. Urine specific gravity was measured using a pocket refractometer (Pocket Pal-10s Refractometer, Atago, Bellevue, WA) by placing one drop of each participant’s urine onto a calibrated refractometer and recording the digital readout. Specific gravity from 1.020 to 1.029 was categorized as mildly dehydrated and 1.030 and above categorized as clinically dehydrated [38,39,40]. 

### 2.5. Data Analysis

Core body temperature data was downloaded using HQ Inc. CorTrak Software and trimmed to exclude biologically implausible numbers that would indicate a body temperature lower than extreme hypothermia or higher than hyperthermia (below 32 °C and above 41 °C, respectively). The percentage of time during the workday that the core body temperature was between 37 to 38 °C and over 38 °C was calculated [41,42]. Descriptive statistics were calculated for the questionnaire responses, WBGT measurements, urine specific gravity, and core body temperatures. Proportions of individuals with specific gravity between 1.020 and 1.030, and above 1.030 were compared using Fisher’s exact test. A Kruskal–Wallis test was used to assess differences in WBGT, specific gravity, and core body temperature by season. Wilcoxon signed rank analysis was performed to compare each season’s post-shift specific gravity to the corresponding morning void. All statistical analyses were completed using STATA 12 (Stata Statistical Software: Release 12, College Station, TX, USA) and R Software (version 3.5.1, Vienna, Austria). An alpha level of 0.05 was considered significant for all statistical tests.

## 3. Results

### 3.1. Study Participants and Questionnaire

A total of 28 participants were recruited and consented over the course of the harvest and post-harvest seasons. The majority of participants listed the Southern state of Chiapas as their home state (*n* = 25 (89.2%)), nearly half spoke an Indigenous language in addition to Spanish (*n* = 12 (42.9%)), most were between the ages of 18 and 24 (*n* = 17 (60.7%)), and none had completed high school (Table 1). Most reported working in agriculture for more than five years (*n* = 17 (60.7%)), working at the current farm for three to five months (*n* = 15 (53.6%)), were contracted by the season (*n* = 20 (71.4%)), paid by the day (*n* = 17 (60.7%)), and worked 10 h per day, seven days per week (*n* = 13 (46.4%) and *n* = 15 ((53.6%), respectively) (Table 2). 

### 3.2. Wet-Bulb Globe Temperature

The WBGT was measured and recorded at multiple points during each sampling day in March, June, and August. Measurements were taken in both the sun and shade, as workers were continually moving in and out of the shade provided by the grape vines. The workers wore standard work pants, long sleeve shirts, caps, and some wore an additional sweater or jacket layer on their top half. 

**Figure 1:**
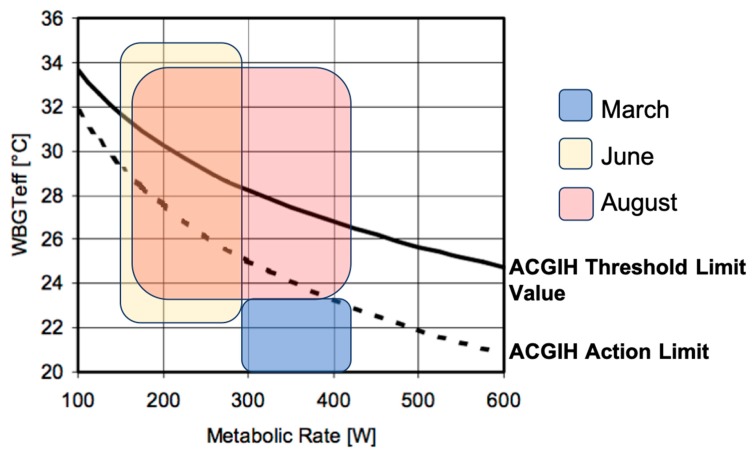
American Conference of Governmental Hygienists Threshold Limit Value and Action Limit for heat stress by month (*n* = 28).

To assess metabolic rate, observations of work activities and work intensity were recorded. In March, the pre-harvest season, the research team observed the workers moving very quickly through the rows of grape vines, trimming and shaping the plant with arms in front of the body, oftentimes stooping low to reach branches or reaching high overhead. The workers frequently ran between rows. As such, the metabolic rate ranged from 300 to 415 W, or moderate to heavy following the ACGIH Metabolic Rate Categories and the Representative Metabolic Rate with Example Activities. The minimum, mean, and maximum WBGT, both in and out of direct sunlight, for March were 13.9 °C, 16.6 °C, and 20 °C, respectively. For all clothing ensembles and metabolic rate combinations, the workers were either below or reaching the ACGIH TLV Action Limit for Heat Stress (Figure 1). 

During the harvest month of June, the research team observed that each worker was responsible for walking down a row of grape vines, cutting off ripe bunches, stacking approximately 10 bunches into a box, and carrying the box to a packaging station. Each worker individually packaged their collected bunches and returned to the row to cut more bunches once packaging was complete. The pace of this work was light to normal. Several workers were responsible for collecting packaged products in wheelbarrows or carts and transporting them to a large trailer nearby. To represent the work completed in June, a metabolic rate range of 180–300 W was used.. A comparison of the WBGT_eff_ to the metabolic rate in Figure 1 reveals that June workers were reaching and exceeding the ACGIH Action Limit and TLV for Heat Stress. The minimum, mean, and maximum WBGT, both in and out of direct sunlight, for June were 22.8 °C, 26.4 °C, and 31.2 °C, respectively.

During the month of August, which was considered the post-harvest season, the research team observed the workers completing a variety of tasks, including: shoveling manure, operating open cab tractors, trimming new grape vines, and other maintenance around the farm as needed. While the fieldwork was very slow paced and required only small movements of the arms and legs, those shoveling were working at a heavy metabolic rate. All WBGT_eff_ reached the ACGIH Action Limit, with mean and maximum temperatures reaching and exceeding the TLV, depending on the clothing ensemble (Figure 1). The minimum, mean, and maximum WBGT, both in and out of direct sunlight, for August were 23.8 °C, 26.5 °C, and 29.7 °C, respectively. The three months (March, June, August) had significantly different WBGT measurements (*p* < 0.01). 

### 3.3. Hydration

**Figure 2:**
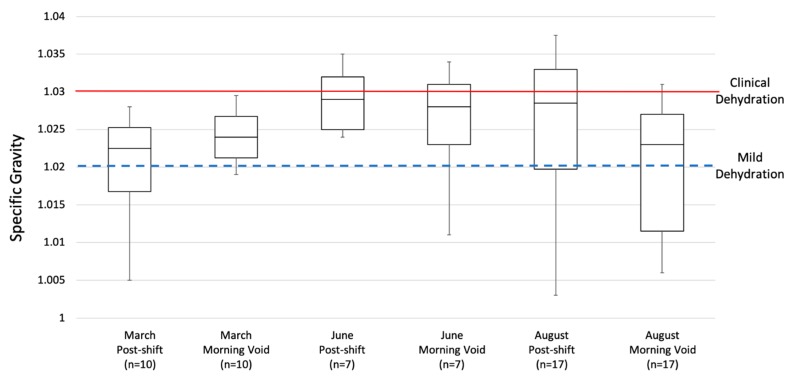
Comparison of specific gravity quartiles by month and collection time (*n* = 28).

As shown in Figure 2, the medians of all urine samples were between 1.020 and 1.030, the cutoffs for mild and clinical dehydration, respectively. The largest percentage of clinical dehydration (S.G. ≥ 1.030) was seen in June morning void samples. The sample with the highest S.G. was collected post-shift in August (S.G. = 1.036), and the sample with the lowest S.G. was also collected post-shift in August (S.G. = 1.003). The mean S.G.s of March post-shift, June post-shift, and August post-shift were 1.25, 1.025, and 1.02, respectively. There was not a statistical difference between months with regards to percent mildly dehydrated or clinically dehydrated. 

### 3.4. Core Body Temperature

**Figure 3:**
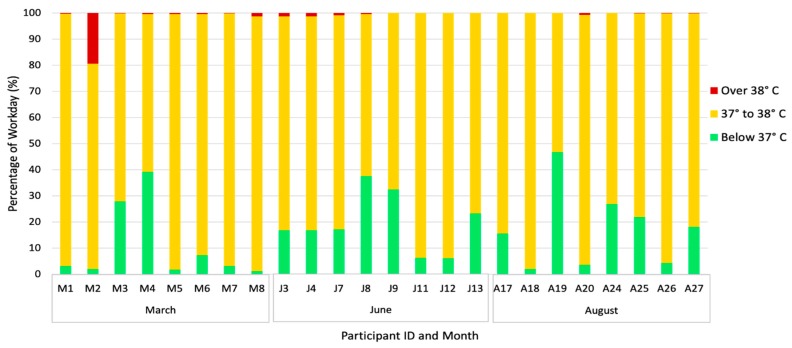
Worker core body temperature during workday, by temperature range (*n* = 24).

The core body temperature of 24 workers was measured, with measurements for eight workers in each month (Table 3). Approximately 3% of the recorded data was either above 41 °C or below 32 °C and was, therefore, removed from further analysis. Workers in March had core body temperatures between 37 and 38 °C for 86.5% of the workday and above 38 °C for 2.3% of the workday (Figure 3). Similarly, workers in June and August had core body temperatures between 37 and 38 °C for 80% and 82.5% of the workday, respectively, with 0.5% and 0.1% of the workday over 38 °C. Workers in March had core body temperatures between 37 and 38 °C and above 38 °C for a longer percentage of their workday than June and August workers, although the difference was not statistically significant. A March worker’s core body temperature remained over 38 °C for the longest percentage of time and an August worker’s core body temperature remained between 37 and 38 °C for the longest percentage of time. The worker in March appears to be an outlier, but there is not a clear indication as to why, given the similarities in work tasks within the March group. There was not a significant difference between mean core body temperatures by month (*p* = 0.26).

## 4. Discussion

The findings from this study suggest that dehydration and heat stress are important and interconnected health outcomes in table grape workers in Sonora, as dehydration status of the body can moderate the response of the body to heat. Workers wore standard work pants, long sleeve shirts, caps, and some wore an additional sweater or jacket layer on their top half during the workday. The metabolic rate ranged from 300 to 415 W in the pre-harvest season during the month of March, with the WBGT_eff_ below the ACGIH Action Limit after incorporating the WBGT and clothing ensemble. During the harvest season in June, worker metabolic rates ranged from 180 to 300 W, surpassing the ACGIH Action Limit and ACGIH TLV with some WBGT and clothing combinations. In August, the post-harvest season, worker metabolic rates ranged from 180 to 415 W, exceeding the ACGIH Action Limit and ACGIH TLV with some WBGT combinations. Hydration status was largely inadequate by the majority of workers during all months. In the most extreme example, over 40% of post-shift urine specific gravities during the month of June were over the threshold for clinically determined dehydration, and almost all others (57%) were considered mildly dehydrated. The levels of dehydration are surprising given the difference in environmental conditions during the three sampling periods, with March, June, and August WBGT measurements averaging WBGTs of 16.6 °C, 26.4 °C, and 26.4 °C, respectively. Workers’ core body temperatures spent little time over the ACGIH TLV of 38 °C during the three sampling events, but did spend at least 80% of the workday between 37 °C and 38 °C.

This project was made possible by the outreach and communication efforts of the University of Arizona and Sonoran research partners over the past seven years. The ability to complete this type of work requires many years of trust and communication to take place; ideally, this farm can set an example for other agricultural entities in Mexico with regards to working and living conditions. It is important to note that the findings from this project are applicable on both sides of the U.S.–Mexico border, as the population, working conditions, migratory status, and climate are very similar. However, the workers included in this study were considerably younger than agricultural workers in studies that have taken place in the U.S. [43,44,45]. 

The research on migrant and seasonal farmworkers that make agricultural production possible in Northern Mexico is limited, especially regarding heat and hydration. This project highlights the need for concentrated efforts to improve hydration regardless of season, further investigate the practice of paying workers by the pound or piece, and implementation of heat stress prevention practices in the agricultural industry. This will require communication and cooperation between farm owners, medical teams, social workers, community health workers, and middle management. Future work should ensure that vocabulary used in written documents is appropriate given the level of education, consider translating into an Indigenous language, and have historical context for Chiapas and internal migration. The use of trained community health workers, usually farmworkers themselves, to communicate environmental and occupational health and hazards has been successfully utilized in agricultural worker populations and may be useful in improving hydration and heat stress outcomes [15]. 

### 4.1. Hydration

Dehydration is commonly reported in agricultural and other physically demanding occupational settings. A study of hydration status at a Costa Rican sugarcane field found that 50% of workers (*n* = 48) were dehydrated [1]. Similar to this study, sugarcane, construction, and farmworkers in Nicaragua had morning urine specific gravities greater than or equal to 1.030 at 15.3%, 28.6%, and 20.4%, respectively [38]. The perception of adequate hydration may not always match reality. As demonstrated in a Nicaraguan sugar farm, highly motivated agricultural workers may lose three to four liters of body water before experiencing extreme thirst [46]. Research in mine settings has demonstrated that workers may not be adequately hydrated in the evenings after their work shifts or in the mornings before their shifts, creating an increased potential for dehydration during the workday [42]. 

We also found that the participants in our study were provided water in the field in the form of large, centrally located mobile water tanks. The water tanks are used to fill personal one-liter water containers that the workers either carry through the field or leave at the beginning of the line. The workers did not readily have access to sports drinks or other electrolyte replacement methods, which is important during high sweat situations [31]. There was a small store near the farm cafeteria that sold other drinks, including soda and sports drinks that were available for purchase but were not allowed in the fields to prevent product contamination. It is important to note that alcohol is forbidden on the farm at all times, but workers were permitted to leave the farm and go into the nearby town and access alcohol, which can contribute to dehydration [47]. Past hydration training by the local university has resulted in increased administrative controls including access to water at all times and the one-liter personal water containers. However, the high turnover rate of agricultural work and elevated urine specific gravities observed in this study demonstrate the need for continuous training and control efforts. With regards to future worker training on hydration, it may be beneficial to incorporate a urine color component to the curriculum, as specific gravity correlates well with urine color and may be used to educate workers on self-assessment of hydration status [48]. The incorporation of additional required water breaks throughout the workday by the management team may also be a useful tool in combating dehydration. 

### 4.2. Heat Stress

We expected to see higher core body temperatures in the summer months when the harvest took place, but the opposite was observed, suggesting that increased core body temperature was more related to metabolic rate than environmental conditions. In March, the workers’ efforts were focused on young grape vines that offered little shade coverage, with workers quickly moving down each line of grapes pruning and shaping the plant and running between lines. There appears to be an inverse relationship between the observed metabolic rate and ambient temperature, suggesting there is decreased activity when temperatures increase and increased activity when temperatures decrease. The intense work pace observed in March may also be a result of how the workers are paid [28,29]. March workers reported being paid by the completion of a row of vines rather than by a set time frame (i.e., hourly, daily, or monthly).

Heat stress has been measured in a variety of agricultural settings in North America using the ACGIH WBGT-workload method, Physiological Strain Index (PSI), and continuous recording of core body temperature. Heat stress, especially during summer months, is a major concern for agricultural populations completing physically demanding work. WBGT measurements in an observational study of 105 sugarcane workers in Costa Rica surpassed the U.S. Occupational Safety and Health Administration recommendations for avoiding heat stress by 9:15 a.m. [1]. In a 2015 study of 46 male and female tree-fruit harvesters in the northwestern U.S., workers were paid by the number of bins collected during the workday, and nearly 75% of workers exceeded the ACGIH Heat Stress Action Limit during the workday in summer months measured via WBGT, suggesting that the payment type played a role in the heat stress exceedance [3]. In a 2014 study of 283 male and female farmworkers in California, U.S., the mean maximum core body temperature sustained for a minimum of 3 min was 38 °C [43]. In this same study, 10 male participants had a PSI of 7.5 or greater, indicating heat strain, using the same ingestible temperature transmitters used in the present study to measure core body temperature [43]. The PSI incorporates heart rate and rectal temperature, or core body temperature, and is contained within a scale of 1–10 [49]. Similarly, over half of 43 participants enrolled in a 2012–2013 fernery worker study in Florida, U.S. exceeded 38 °C at some point during the workday, with one worker’s core body temperature reaching 38.9 °C [45]. 

It is important to note that the on-site dormitories the workers sleep in are single-layer cinderblock structures, with two to three cinderblock sleeping bunks stacked vertically. Previous projects carried out by the research team revealed an average of eight square feet per person of floor space in the dormitories. The crowded dormitories rely on natural ventilation for airflow, including open doors and large cutouts near the top of the structures. During a summer 2015 housing assessment conducted by the research team, the temperature inside the dormitories exceeded 32 °C during the day, which may prevent workers from adequately cooling down when they are inside the dormitories overnight and when they are not working during the day [34]. 

### 4.3. Limitations

There were several limitations of this project that should be recognized to inform future endeavors. As with many other pilot projects, the small sample size limits the statistical power of any comparisons that are made. For recruitment purposes, we relied heavily on the assistance of the farm doctors and engineering teams to recruit participants for the study, making the sample recruitment partially driven by convenience. Additionally, some of the recruitment took place by word of mouth from worker to worker. This may have biased the type of workers that were included in the study. The heat assessment may have been strengthened by the following: measuring heart rate throughout the day to be able to calculate the PSI; asking about caffeine, diuretics, acetaminophen, and alcohol consumption; and measuring workers’ pre- and post-shift body weight. As mentioned previously, the metabolic rate and clothing ensemble estimations used in the WBGT calculation rely on subjective observations made by the research team; future endeavors should include an objective measure of energy expenditure, such as a continuously monitoring accelerometer or heart rate monitor. Unfortunately, we did not include the rate of temperature rise and response to rest in this manuscript as individual workers were not observed the entire duration of wearing the sensor. Therefore, we did not have a concrete record of their periods of rest and work. We hope to include this data collection and analysis in future endeavors. In all, this pilot study has helped uncover necessary measures and precautions that will be useful in future work.

## 5. Conclusions

This project gives insight into the demographics, work practices, and occupational exposures of a potentially vulnerable and underserved population. Dehydration was measured (urine S.G. >1.020) in the urine of the majority of workers during all seasons, the mean urinary S.G.s of March post-shift, June post-shift, and August post-shift were 1.25, 1.025, and 1.02, respectively. The estimated WBGT_eff_ in the harvest and post-harvest seasons exceeded the ACGIH TLV for heat stress. The percentage of time workers’ core body temperatures were over the ACGIH TLV of 38 °C was minimal for most workers during the pre-harvest, harvest, and post-harvest seasons. However, core body temperatures between 37 °C and 38 °C made up over 80% of the workdays. As the number of warm days is expected to increase each year, it will be increasingly important to engage in preventive practices to protect vulnerable populations, such as migratory and seasonal agricultural workers. 

## Figures and Tables

**Table 1:** Participant demographic information by season.

	All Seasons (*n* = 28)	Harvest Season (*n* = 11)	Post-Harvest Season (*n* = 17)
	*n* (%)		
Mexican state of origin			
Chiapas	25 (89.2)	10 (90.9)	15 (88.2)
Tabasco	1 (3.6)	1 (9.1)	0
Aguascalientes	1 (3.6)	0	1 (5.9)
Veracruz	1 (3.6)	0	1 (5.9)
Speak an Indigenous language			
Yes	12 (42.9)	1 (9.1)	11 (64.7)
No	16 (57.1)	10 (90.9)	6 (35.3)
Age (years)			
18–24	17 (60.7)	3 (27.3)	14 (82.4)
25–30	7 (25.0)	6 (54.5)	1 (5.9)
>30	4 (14.2)	2 (18.2)	2 (11.8)
Highest level of education completed			
None	4 (14.3)	1 (9.1)	3 (17.6)
Elementary school and some junior high (Grades 1–7)	17 (60.7)	5 (45.5)	11 (64.7)
Junior high and some high school (Grades 8–12)	7 (25.0)	4 (36.4)	3 (17.6)
All high school	0 (0.0)	0 (0.0)	0 (0.0)

**Table 2:** Participant self-reported work details by season.

	All Seasons (*n* = 28)	Harvest Season (*n* = 11)	Post-Harvest Season (*n* = 17)
	*n* (%)		
Total length of time spent working in agricultural industry			
Less than 3 months	1 (3.6)	0 (0.0)	1 (5.9)
3–5 months	1 (3.6)	1 (9.1)	0 (0.0)
6 months to 1 year	3 (10.7)	1 (9.1)	2 (11.8)
1 to 5 years	6 (21.5)	2 (18.2)	4 (23.6)
>5 years	17 (60.7)	7 (63.6)	10 (58.9)
Length of time at current farm			
Less than 3 months	3 (10.7)	1 (9.1)	2 (11.8)
3–5 months	15 (53.6)	8 (72.7)	7 (41.2)
6 months to 1 year	1 (3.6)	0 (0.0)	1 (5.9)
1 to 5 years	3 (10.7)	1 (9.1)	2 (11.8)
>5 years	6 (21.5)	1 (9.1)	5 (29.4)
Contract type			
By year	8 (28.6)	0 (0.0)	8 (47.1)
By season	20 (71.4)	11 (100.0)	9 (52.9)
Payment type			
By the day	17 (60.7)	2 (18.2)	15 (88.2)
By the month	1 (3.6)	0 (0.0)	1 (5.9)
By the pound/piece	10 (35.7)	9 (81.8)	1 (5.9)
Hours worked per day			
7	6 (21.4)	1 (9.1)	5 (29.4)
8	6 (21.4)	1 (9.1)	5 (29.4)
9	0 (0.0)	0 (0.0)	0 (0.0)
10	13 (46.4)	9 (81.8)	4 (23.6)
>10	3 (10.7)	0 (0.0)	3 (17.6)
Days worked per week			
5	0 (0.0)	0 (0.0)	0 (0.0)
6	13 (46.4)	1 (9.1)	12 (70.6)
7	15 (53.6)	10 (90.9)	5 (29.4)

**Table 3:** Core body temperature by participant and month, *n* = 24.

Month (Season)	Subject ID	Minimum (°C)	Maximum (°C)	Median (°C)	Mean (°C)	Standard Deviation	Time Spent 37 to 38 °C (%)	Time Spent Over 38 °C (%)
March (Pre-Harvest)	M1	31.4	39.9	37.4	37.3	0.4	96.5	0.3
	M2	28.2	38.2	37.6	37.6	0.5	78.6	19.4
	M3	27.0	39.5	37.2	37.1	0.7	72.0	0.2
	M4	28.2	39.9	37.1	36.8	1.3	60.4	0.4
	M5	28.8	39.4	37.4	37.4	0.4	97.9	0.4
	M6	31.3	39.5	37.4	37.4	0.4	92.3	0.4
	M7	27.8	38.8	37.6	37.5	0.4	96.7	0.2
	M8	27.9	39.6	37.4	37.3	0.5	97.6	1.3
	**Overall** **Mean**				**37.3**	**0.6**	**86.5**	**2.3**
June (Harvest)	J3	26.9	40.2	37.3	37.1	1.2	81.8	1.3
	J4	26.9	40.2	37.3	37.1	1.2	81.8	1.3
	J7	28.2	39.6	37.2	37.2	0.5	82.1	0.8
	J8	27.7	39.5	37.1	37.0	0.6	62.0	0.4
	J9	27.1	37.8	37.1	37.0	0.8	67.5	0.0
	J11	30.5	37.6	37.4	37.3	0.3	93.7	0.0
	J12	27.0	37.9	37.5	37.4	0.6	93.8	0.0
	J13	36.1	37.9	37.2	37.2	0.2	76.7	0.0
	**Overall** **Mean**				**37.2**	**0.8**	**80.0**	**0.5**
August (Post-Harvest)	A17	32.2	39.0	37.5	37.3	0.4	84.4	0.0
	A18	32.0	37.9	37.5	37.4	0.4	98.0	0.0
	A19	32.6	38.7	37.0	36.9	0.4	53.3	0.0
	A20	31.3	40.2	37.5	37.4	0.4	95.7	0.7
	A24	31.2	37.7	37.2	37.1	0.4	73.1	0.0
	A25	31.6	38.1	37.3	37.2	0.5	78.1	0.1
	A26	31.7	38.1	37.5	37.4	0.3	95.6	0.1
	A27	30.7	39.9	37.2	37.1	0.5	81.6	0.2
	**Overall** **Mean**				**37.2**	**0.4**	**82.5**	**0.1**
